# Teriparatide-Associated Hypercalcemia Concomitant With Acute Kidney Injury: A Case-Report

**DOI:** 10.7759/cureus.54263

**Published:** 2024-02-15

**Authors:** Yuya Goto, Yuri Uchiyama, Tomoyuki Fujikura, Takeshi Tashiro, Hideo Yasuda

**Affiliations:** 1 Internal Medicine 1, Hamamatsu University School of Medicine, Shizuoka, JPN

**Keywords:** osteoporosis, immobilization, acute kidney injury, persistent hypercalcemia, teriparatide

## Abstract

Teriparatide, a recombinant human parathyroid hormone, is an anabolic treatment for osteoporosis with a high risk of fractures. Transient hypercalcemia is an adverse effect of teriparatide and usually resolves within 16h of teriparatide administration owing to its rapid absorption and elimination. Some cases of prolonged hypercalcemia have been reported, but these improved rapidly after teriparatide discontinuation. Here, we describe a rare case of teriparatide-induced hypercalcemia concomitant with acute kidney injury that persisted for four weeks. An 83-year-old woman began taking teriparatide for a vertebral fracture. The patient was immobilized by the fracture. Three weeks later, the patient developed hypercalcemia and acute kidney injury. However, hypercalcemia persisted for four weeks despite the discontinuation of teriparatide and fluid administration. Clinicians should be aware that teriparatide can induce severe hypercalcemia, especially in the setting of immobilization, and that hypercalcemia can persist for more than 3-4 weeks in patients with decreased kidney function.

## Introduction

Most treatments for osteoporosis (e.g., bisphosphonates and Denosumab) are antiresorptive agents that inhibit bone turnover and decrease bone resorption. Teriparatide, an anabolic agent that stimulates bone turnover, is a recombinant human parathyroid hormone (PTH). Transient hypercalcemia due to teriparatide may occur; however, serum calcium levels are maximal at 4-6 h after teriparatide administration and return to baseline within 24 h [1] because of its rapid absorption and elimination. Although some patients showed hypercalcemia, their condition improved rapidly after the discontinuation of teriparatide [2]. To date, hypercalcemia sustained for more than a few weeks after teriparatide discontinuation has not yet been reported. We encountered a patient who developed teriparatide-associated hypercalcemia concomitant with acute kidney injury, which sustained for 4 weeks even after discontinuation of teriparatide.

## Case presentation

**Figure 1:**
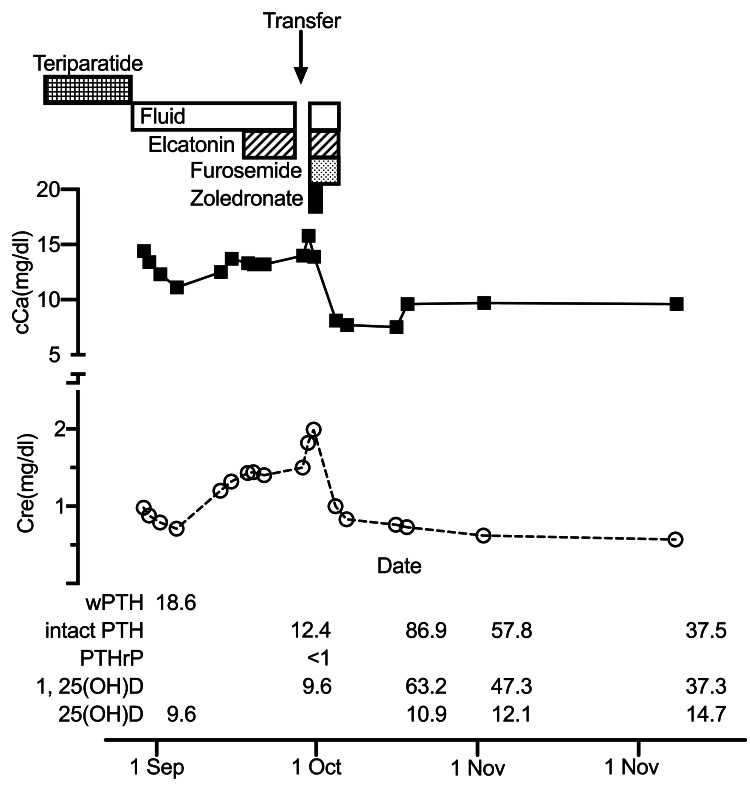
Summary of the clinical curse, including Ca and Cre serum levels. Cessation of teriparatide followed by other treatments is depicted. cCa: Corrected calcium (mg/dl), Cre: Creatinine (mg/dl), wPTH: whole PTH(1-84) (pg/ml), iPTH: intact PTH (pg/ml), PTHrP: PTH related peptide (pmol/l), 1, 25(OH)D: 1,25-dihydroxyvitamin D 3 (ng/ml), 25(OH)D: 25-dihydroxyvitamin D 3 ng/ml

An 83-year-old woman was diagnosed with a compression fracture of the thoracic vertebra, and subcutaneous teriparatide 20 µg/day was started. Eleven days after the initiation of teriparatide treatment, the patient presented to the hospital with persistent back pain. She had hypercalcemia with a calcium level of 14.4 mg/dL and was admitted to the hospital. Her past medical history included bronchial asthma and hypertension. She was taking magnesium oxide, lansoprazole, zolpidem tartrate, tramadol hydrochloride, acetaminophen, amlodipine besilate, and clopidogrel sulfate. She was not taking oral corticosteroids, vitamin D, or calcium supplements. The patient was in bed because of back pain and was unable to move without assistance. The patient had diffuse muscle weakness. Laboratory work-up, including phosphorus, 25-hydroxyvitamin D, PTH-related peptide (PTHrP), and thyroid-stimulating hormone levels, was unremarkable. Spinal magnetic resonance imaging did not reveal any masses. Treatment with teriparatide was discontinued, and intravenous fluid, furosemide, and elcatonin injections were initiated. However, four weeks after admission, she developed persistent hypercalcemia and was transferred to our hospital for further management. The blood tests revealed that her creatinine was 1.82 mg/dL, calcium was 15.7 mg/dL, phosphorus was 4.5 mg/dL, intact PTH (iPTH) was 12.4 ng/L, 1,25(OH)2 vitamin D (calcitriol) was 9.6 pg/mL, and PTHrP was <1.0 pmol/L. Computed tomography and electrophoresis results were normal. Initial treatment consisted of 0.9% isotonic intravenous saline, furosemide, zoledronic acid, and elcatonin. Hypercalcemia and decreased kidney function improved 5 days after treatment. The patient was discharged 10 days after admission, and her serum calcium and creatinine levels remained normal during the subsequent follow-ups (Fig 1).

## Discussion

We present a case of hypercalcemia that highlights two clinically important issues. First, teriparatide can also cause severe hypercalcemia, especially in the setting of immobilization. Second, concomitant with acute kidney injury, hypercalcemia can persist for four weeks even after discontinuation of teriparatide.

Because of the rapid absorption and elimination of teriparatide, serum calcium concentration increases maximally at 4.25 h and then returns to the baseline concentration by 16-24h [1]. Transient hypercalcemia is an uncommon adverse complication of teriparatide and is reported in only 1-3% of patients [3]. Several cases of hypercalcemia due to teriparatide have been reported, most of which resolved within a few days after teriparatide discontinuation [4]. Although the pathophysiology is not fully understood, we believe that the onset of hypercalcemia in our patient was associated with increased bone resorption. Immobilization can cause hypercalcemia even in adults. Adult cases of hypercalcemia due to immobilization due to spinal fractures [5], spinal cord injury [6], and critical illness due to coronavirus disease 2019 have [7] been reported. Immobilization-induced sudden loss of mechanical forces in bones is thought to increase osteoclastic bone resorption and decrease osteoblastic bone formation, which could contribute to the onset of hypercalcemia [6].

Additionally, high bone turnover may accelerate immobilization-induced hypercalcemia. Children and adolescents are more vulnerable to immobilization-induced hypercalcemia because of their high bone turnover rates [8]. Teriparatide stimulates bone formation and resorption, resulting in high bone turnover [9]. Therefore, we speculated that teriparatide-induced high bone turnover accelerates immobilization-induced hypercalcemia.

Second, hypercalcemia can persist for four weeks, concomitant with acute kidney injury. Decreased kidney function contributes to persistent hypercalcemia. A vicious circle between hypercalcemia and decreased kidney function causes this. Hypercalcemia leads to decreased kidney function through a combination of natriuretic effects followed by hypovolemia and renal vasoconstriction [10,11]. Decreased kidney function limits the ability of the kidneys to excrete calcium, leading to persistent hypercalcemia. A case of hypercalcemia due to vitamin D intoxication concomitant with acute kidney injury was reported. In this case, hypercalcemia persisted for four weeks even after discontinuation of vitamin D and administration of fluids, and treatment with Denosumab was needed [12]. Because of this vicious circle, attention should be paid not only to hypercalcemia but also to kidney function to prevent persistent hypercalcemia.

## Conclusions

We encountered a case of teriparatide-associated hypercalcemia resulting from immobility due to a vertebral fracture that persisted for four weeks because of acute kidney injury. It is important to note that teriparatide, even if rare, can cause severe hypercalcemia and that immobilized patients in the acute phase of bone fractures have a higher risk of hypercalcemia. We also need to recognize that a vicious cycle between hypercalcemia and decreased kidney function prolongs the duration of hypercalcemia, and we should follow them up for more than a few weeks.
